# Effectiveness of Distance Educational Programs for parents of children diagnosed with Autism Spectrum Disorder: an integrative review

**DOI:** 10.1590/2317-1782/20242023291en

**Published:** 2024-09-02

**Authors:** Jullie Gottschall Lima Andrade, Andreia Cristina Feitosa do Carmo, Ana Carina Tamanaha, Jacy Perissinoto

**Affiliations:** 1 Departamento de Fonoaudiologia, Escola Paulista de Medicina, Universidade de São Paulo – UNIFESP - São Paulo (SP), Brasil.; 2 Programa de Pós-graduação Distúrbios da Comunicação Humana, Universidade de São Paulo – UNIFESP - São Paulo (SP), Brasil.; 3 Núcleo de Investigação Fonoaudiológica da Linguagem da Criança e Adolescente no TEA, Escola Paulista de Medicina, Universidade de São Paulo – UNIFESP - São Paulo (SP) , Brasil.

**Keywords:** Autism, Telemedicine, Child Development, Parents, Autism Spectrum Disorder

## Abstract

**Objective:**

to analyze the scientific literature on distance education programs for parents/caregivers in the development of children with Autism Spectrum Disorder (ASD).

**Research Method:**

the PICO strategy was used to identify the research problem. The databases Medline, ERIC, LILACs, EMBASE, CINAHL, Web of Science, and Scopus were searched using specific descriptors and free terms. There were no restrictions on time or language. Articles on online educational programs for parents of children with ASD were selected, focusing on the impact of these programs on the development of children up to six years old.

**Selection Criteria:**

studies were selected based on standard eligibility criteria, including full-text reading after initial screening using the RAYYAN software. Primary studies such as clinical trials and systematic reviews evaluating distance education programs for parents of children with ASD were included.

**Data Analysis:**

the RAYYAN software was used for initial study selection. Articles were hierarchically organized based on title and abstract, followed by full-text reading to apply eligibility criteria.

**Results:**

the initial search yielded 1019 articles, of which 192 were identified as duplicates. After initial screening and full-text reading, 37 articles were analyzed, of which six were deemed eligible to answer the research question. Among the eligible studies, one was a systematic review and five were experimental studies. Experimental studies highlighted positive impacts on areas such as daily routines, behavioral flexibility, and communication. The systematic review provided preliminary evidence that distance education programs for parents can enhance knowledge about ASD, increase adherence to interventions, and foster the development of social and communication skills in children.

**Conclusion:**

the findings suggest that remote parent guidance programs may effectively improve knowledge about ASD, increase parent adherence to interventions, and promote the development of social and communication skills in children with ASD.

## INTRODUCTION

Autism Spectrum Disorder (ASD) is a neurological condition that manifests in childhood and persists throughout life. It is characterized by persistent deficits in social interaction and communication, impairments in socioemotional reciprocity, and restricted and repetitive patterns of behavior, interests, or activities^(1)^. ASD is a complex, pervasive, and heterogeneous disorder^(2)^, affecting multiple areas of development and presenting extremely variable characteristics among affected individuals. This variability is partly due to the wide range of genes and environmental factors^(3)^ that can contribute to the development of ASD. The complexity of this disorder represents a significant challenge for individuals, families, and healthcare systems.

Given the complex nature of ASD, early, multidisciplinary, and long-term intervention is vital^(4)^. Early intervention involves a combination of specialized therapies, including behavioral therapy, speech therapy, occupational therapy, and, in some cases, medication^(5)^.

Due to the great heterogeneity among children with ASD, it is necessary to choose interventions that make sense for each individual and adjust treatment expectations, as responses to treatment can vary considerably. According to the CDC^(6)^, one in every 36 children aged 8 years old in the United States is diagnosed with autism, representing 2.8% of children population. This high prevalence presents significant challenges for the healthcare network and families, given the shortage of trained professionals and the concentration of referral centers in central regions. In geographically large countries like Brazil, access to treatments and interventions can be particularly challenging in peripheral, inland, and rural areas^(7)^. This highlights the need to create intervention alternatives that are not only effective but also accessible and scalable.

In the field of Speech-Language Pathology, the practice of Tele-Speech-Language Pathology was regulated by RESOLUTION CFFa No. 580, dated August 20, 2020. This includes synchronous and asynchronous practices, as well as teleconsultations, telemonitoring, and providing a basis for distance parental guidance practices using technology.

Early intervention, while beneficial, can be costly for families and the state, making it necessary to create different forms of intervention. Recent studies have shown the effectiveness of using technology to conduct remote interventions^(8)^, which can help bridge this gap. However, it is important to note that remote intervention modalities may disproportionately benefit those with higher socioeconomic status, highlighting the need to ensure equity in access to these resources^(9)^.

Among remote intervention modalities, parent education and training have shown significant gains in the language development of children with ASD. The literature provides evidence^(10-12)^ that difficulties or delays in children's communication are associated with changes in parental behavior.

With the constant evolution of technology, online educational programs for parents and caregivers can offer interactive learning modules, support resources, and expert guidance to address specific issues related to ASD^(13)^. These programs can range from training on specific behavioral techniques to guidance on navigating education and healthcare systems to ensure the appropriate rights and services for these children. However, the potential benefits of these programs must be weighed against the associated challenges. For example, some families may have difficulty accessing reliable internet or may not be familiar with the required technology to participate in these programs. Additionally, although many online programs are designed to be self-guided, some parents may feel overwhelmed without direct support from a professional^(14)^.

Given the diversity of experiences and contexts of individuals with ASD and their families, it is essential that remote educational programs are flexible and customizable to meet the specific needs of each family. Furthermore, research should continue to evaluate the effectiveness of these programs in different populations and contexts to ensure that they are a useful and accessible tool to support the development of children with ASD.

Therefore, it is necessary to understand and synthesize the current state of scientific knowledge regarding the impact and understanding of the effectiveness and applicability of remote educational programs for parents and caregivers of children diagnosed with ASD. Based on the results, we will recommend strategies to improve the implementation of these programs, ensuring they broadly and equitably benefit all families facing the challenges associated with ASD.

## METHOD

This study consists of an integrative literature review^(15)^, a method that allows the combination of empirical and theoretical studies to search for broader evidence on a specific topic. Due to the inclusive nature of this type of review, it is possible to address a wider range of purposes, such as concept definition, theory review, evidence review, and methodological practice analysis^(16)^.

Our integrative review followed a structured six-step procedure^(17)^: (1) preparation of the research question; (2) definition of descriptors and keywords; (3) selection of articles according to eligibility criteria; (4) data collection, extraction, reading, and critical analysis of the articles; (5) interpretation and discussion of the results; (6) knowledge synthesis and presentation of the review^(18)^.

To formulate the guiding question, we used the PICO strategy^(17,19,20)^ (Patient, Intervention, Comparison, Outcomes), a useful tool for structuring health research questions. In this case, the first element (P) referred to caregivers or family members of children with autism; the second (I), to distance educational programs for these caregivers; the third element (C) was not used in this review; and the fourth element (O) refers to the skills, language development, and cognitive development of the children.

Thus, the guiding question of this review was: "What are the impacts of distance educational programs for caregivers or family members of children diagnosed with Autism Spectrum Disorders?"

The search for relevant studies was conducted in December 2021 in the databases Medline, ERIC, LILACs, EMBASE, CINAHL, Web of Science, and Scopus. We used a combination of keywords and descriptors selected from Medical Subject Headings (MeSH), Emtree Terms, and Health Sciences Descriptors (DeCS) from the Virtual Health Library (VHL) (Table 1).

**Table 1:** Search strategy for the databases

DATABASE	STRATEGY	Qty.
PUBMED	("parents"[MeSH Terms] OR ("caregiver s"[All Fields] OR "caregivers"[MeSH Terms] OR "caregivers"[All Fields] OR "caregiver"[All Fields] OR "caregiving"[All Fields]) OR "parents"[Text Word] OR "family relations"[MeSH Terms] OR "single mother"[Text Word]) AND ("autism spectrum disorder"[MeSH Terms] OR "autistic disorder"[MeSH Terms]) AND ("education, distance"[MeSH Terms] OR ("education"[All Fields] AND "distance"[All Fields]) OR "distance education"[All Fields] OR ("distance"[All Fields] AND "education"[All Fields]) OR "e-learning"[All Fields] OR ("internet"[MeSH Terms] OR "internet"[All Fields] OR "internet s"[All Fields] OR "internets"[All Fields]) OR ("computability"[All Fields] OR "computable"[All Fields] OR "computating"[All Fields] OR "computation"[All Fields] OR "computational"[All Fields] OR "computations"[All Fields] OR "compute"[All Fields] OR "computed"[All Fields] OR "computer s"[All Fields] OR "computers"[MeSH Terms] OR "computers"[All Fields] OR "computer"[All Fields] OR "computes"[All Fields] OR "computing"[All Fields] OR "computional"[All Fields]) OR "internet based intervention"[MeSH Terms] OR "on line intervention"[Text Word])	221
ERIC	(caregivers OR "family relationship" OR "child parent relation" OR “parenting skills”) AND (autism OR “autistic disorders” OR “autistic children” OR “autism spectrum disorders”) AND (“distance education” OR “distance learning” OR tele-education OR "visual classroom" OR e-learnings OR "internet-based intervention" OR "web-based intervention" OR internet) AND ("parent education") AND adult education	488
EMBASE	('child parent relation'/exp OR 'child parent relation' OR 'child parent relationship' OR 'child parent spatial pattern' OR 'correlation, parent child' OR 'parent child correlation' OR 'parent child relation' OR 'parent child relationship' OR 'parent infant bonding' OR 'parent infant relation' OR 'parent-child relations' OR 'parental role' OR 'parenting' OR 'parent'/exp OR 'biological parent' OR 'parent' OR 'parents' OR 'single parent'/exp OR 'lone parent' OR 'mother, unmarried' OR 'parent, single' OR 'single parent' OR 'unmarried mother' OR 'unwed mother' OR 'family relation'/exp OR 'family relation' OR 'family relations' OR 'caregiver'/exp OR 'care giver' OR 'caregiver' OR 'caregivers' OR 'carer' OR 'carers' OR 'family caregiver' OR 'family caregivers') AND ('autism'/exp OR 'kanner syndrome' OR 'pdd (pervasive developmental disorder)' OR 'autism' OR 'autism spectrum disorder' OR 'autism, early infantile' OR 'autism, infantile' OR 'autistic child' OR 'autistic children' OR 'autistic disorder' OR 'autistic spectrum disorder' OR 'child development disorders, pervasive' OR 'childhood autism' OR 'classical autism' OR 'early infantile autism' OR 'infantile autism' OR 'infantile autism, early' OR 'pervasive child development disorders' OR 'pervasive developmental disorder' OR 'pervasive developmental disorders' OR 'typical autism') AND ('distance learning'/exp OR 'distance education' OR 'distance learning' OR 'education, distance' OR 'tele-education' OR 'teleeducation' OR 'virtual classroom' OR 'virtual education' OR 'e learning'/exp OR 'e-learning' OR 'e-schooling' OR 'electronic education' OR 'electronic educational technology' OR 'electronic learning' OR 'on-line education' OR 'on-line learning' OR 'online education' OR 'online learning' OR 'online schooling' OR 'web-based intervention'/exp OR 'internet-based intervention' OR 'internet-intervention' OR 'online-based intervention' OR 'online-intervention' OR 'web intervention' OR 'web-based intervention' OR 'telehealth'/exp OR 'e-health' OR 'ehealth' OR 'tele-health' OR 'telehealth')	187
LILAC’s	("distance education" OR "educacao a distancia"OR e-leraning OR tele-educac* OR internet OR "web basead intervention" OR "online education" OR "educacao online") AND (autism* OR autisti*) AND (pais OR maes OR parentes OR familia* OR cuidador*)	132
CINAHL	("parent education" OR caregivers OR "family relationship" OR "child parent relation" OR "parenting skills") AND (autism OR "autistic disorders" OR "autistic children" OR "autism spectrum disorders") AND ("distance education" OR "distance learning" OR “tele-education” OR "visual classroom" OR “e-learnings” OR "internet-based intervention" OR "web-based intervention" OR internet)	
Web of Science	("parent education" OR caregivers OR "family relationship" OR "child parent relation" OR "parenting skills") AND (autism OR "autistic disorders" OR "autistic children" OR "autism spectrum disorders") AND ("distance education" OR "distance learning" OR “tele-education” OR "visual classroom" OR “e-learnings” OR "internet-based intervention" OR "web-based intervention" OR internet)	40
SCOPUS	(“parent education" OR caregivers OR "family relationship" OR "child parent relation" OR "parenting skills") AND (autism OR "autistic disorders" OR "autistic children" OR "autism spectrum disorders") AND ("distance education" OR "distance learning" OR “tele-education” OR "visual classroom" OR “e-learnings” OR "internet-based intervention" OR "web-based intervention" OR internet)	123

The search strategy was developed with the contribution of an information professional with extensive experience in the field. Additionally, the recommendation PRESS - Peer Review of Electronic Search Strategies^(21)^ was adhered to the search and identification process, ensuring the quality, systematization, and transparency of the search strategy construction process. There was no date restriction, aiming for a comprehensive and historical understanding of the topic.

Inclusion criteria considered online educational programs focused on teaching parents and/or caregivers of children diagnosed with Autism Spectrum Disorder (ASD) under six years old. Exclusion criteria included educational programs used to complement therapies, those addressing direct intervention for children, and studies that did not discuss the impact on children's skills after the intervention. Programs exclusively for skills assessment or diagnosis were also excluded.

After the database search, the software Rayyan^(22)^ was used to manage the selection of primary studies. For data analysis, duplicates were initially excluded by the software itself. Subsequently, two independent reviewers conducted the analysis, classifying the articles as "included," "maybe," and "excluded."

After the final selection of studies, data extraction was carried out. To ensure consistency and accuracy in this process, a data extraction form was developed, including information on the author, year of publication, country of origin, study objective, study design, target population, intervention (if applicable), main results, and conclusions. This data extraction process was conducted independently by two reviewers. Each filled out the data extraction form for each selected study. After extraction, both reviewers met to discuss and resolve any discrepancies in the extracted information. In cases of disagreement, a third reviewer was consulted to make the final decision. The extracted data were then synthesized and organized to answer the research question. The data extraction process was essential to ensure a complete understanding of the content of each study, enabling comparative analysis and synthesis of all included studies.

## RESULTS

**Figure 1:**
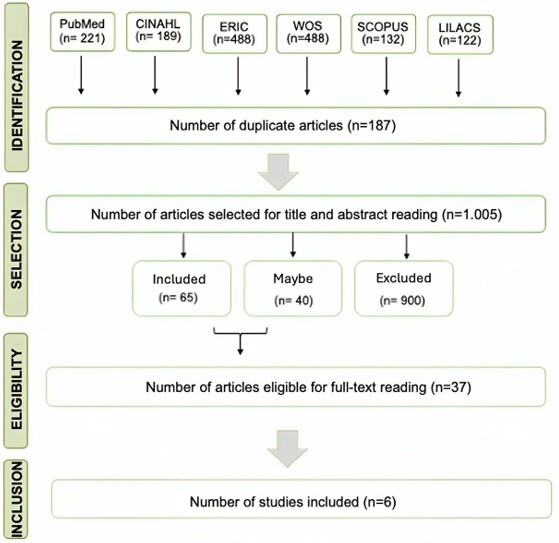
Flowchart of study selection

The initial search identified 1,192 studies, of which thirty-seven were selected for full-text reading. After applying the eligibility criteria, six studies were included for analysis, as illustrated in Figure 1.

The selected articles were published between 2017 and 2021, with three articles from the United States^(23-25)^, one from Australia^(26)^, one from Iceland^(27)^, and one from Japan^(28)^. The results analysis was conducted descriptively, considering the variables of interest for this review. Detailed information on each study is presented in Table 1.

The samples of the studies were heterogeneous, ranging from 3 parents (in a single case study) to 104 parents of children with ASD. In all experimental studies, an online methodology and phased programs were used.

Regarding the data analysis of the experimental studies, it was noted that all were divided into three or four phases, including in-person, online, or mixed methods. In the in-person and synchronous online phases, guidance occurred individually with each family. Additionally, the sequence of phases was very similar, even with different terminologies.

The programs described in the articles demonstrated positive impacts on the development of the children. The eligible articles responded to the guiding question, indicating that distance education for parents is a viable and effective alternative for impacting children's development (Descriptive Chart 1).

**Chart 1:** Data from the studies included in this review

Author	Year	Location	Objectives	Participants	Study Type	Method	Main Outcomes
Guðmundsdóttir et al.^(27)^	2018	Iceland	Develop and evaluate an effective telehealth procedure for skill gains in children with ASD.	3	Single-case experimental	Mixed: face-to-face and synchronous online	Family 1: Training was not effective for increasing sentence length, but was effective for increasing social responsiveness. Family 2: Highly effective for social responsiveness and requests. Family 3: Effective for social responsiveness and ineffective for sentence length.
Ibañez et al.^(23)^	2018	USA	Examine the effectiveness of an interactive web-based parental tutorial to enhance children with ASD's engagement in daily routines.	104 = Tutorial group (n = 52) or Control group (n = 52)	Randomized controlled trial	Online - Asynchronous	The tutorial group showed gains in daily routines, changed routine-related behaviors, and achieved broad outcomes for both children and parents, such as improvement in social communication and reduction in problem behaviors. Children and their parents were able to progress through lower levels of participation (i.e., tolerant) and reached higher levels of participation.
Douglas et al.^(24)^	2017	USA	Evaluate the effect of an online training for parents on the communication skills of children with ASD.	3	Single-case experimental	Mixed: face-to-face and online - Asynchronous	In terms of communication opportunity skill: moderate effect. In communication: Family 1 and 3 showed moderate effects and Family 2 showed strong effects. Parental response: Families 1 and 3 exhibited high effects, and Family 2 showed strong effects.
Hong et al.^(28)^	2018	Japan	Evaluate the effectiveness of an online training package in language development for children with ASD and communication deficits.	3	Single-case experimental	Online - Asynchronous	One of the families dropped out of the study due to difficulty with technology. Family 1: There was a substantial increase in communication skills; Family 2: There was a moderate increase; In specific communication objectives: Family 1 showed an immediate increase in the number of communication opportunities provided; Family 2 showed a consistent increase in the number of different words used.
Kunze et al.^(25)^	2021	USA	Evaluate the effectiveness of an online parent training program in reducing inflexible behaviors in children with ASD.	6	Single-case experimental	Mixed: face-to-face and online - Synchronous and Asynchronous	Family 1 dropped out due to an incident related to the pandemic; there was improvement in flexible behaviors and a decrease in inflexible behaviors in all families.
Parsons et al.^(26)^	2017	Australia	Developed and evaluated an effective telehealth procedure for skill gains in children with ASD. An effective telehealth procedure for skill gains in children with ASD	3	Systematic Review	Mixed: face-to-face and synchronous online	Family 1: Training was not effective for increasing sentence length but was effective for increasing social responsiveness. Family 2: Highly effective for social responsiveness and requests. Family 3: Effective for social responsiveness and ineffective for sentence length.

## DISCUSSION

The purpose of this integrative review was to identify and analyze the main results found in published articles on the impact of distance educational programs for parents/caregivers on the development of children diagnosed with ASD. Our analysis demonstrated that parental guidance is a promising intervention model for early intervention in this group of children.

The six articles reviewed in this research^(23-28)^ corroborate the efficacy of distance intervention, observing significant parental adherence to the intervention program and improvements in children's development.

From the experimental articles, a diversity of methodologies was noted, highlighting the need for future research to determine more systematized practices aiming at ease of replication in clinical practice. Another point that differs among the programs is the delivery mode of the educational program—synchronous or asynchronous. However, most studies (three out of five experimental studies) combined both modalities.

The systematic review^(26)^ found in this article indicates that more interactive delivery methods, with videos and regular contact with the therapist, demonstrate improvements in adherence, increased completion rates, and better adherence to parent-mediated interventions. Hence, the combination of modalities may be more effective, offering more possibilities for the parents' learning process.

The experimental studies commonly featured phased intervention programs, with very similar phase sequences, albeit with different terminologies. Among the study phases, most included a pre-intervention phase, a training phase, and a post-intervention phase.

The training phase, characterized by teaching parents to perform the intervention with the children on the target skills, was the most different among the studies, but most used the asynchronous method (three out of five studies).

The literature discusses that the advantages of asynchronous care include reducing participation impact due to technological issues, such as connectivity observed in synchronous intervention. This service increases access, care quality, and reduces care costs. Additionally, asynchronous intervention presents a possible solution for workforce shortages and a strategy to improve existing care systems^(29)^.

However, the advantage of the synchronous model is the possibility of providing immediate feedback, suggesting it is more effective in producing behavioral changes^(30)^. The synchronous practice in the findings of this review ^(25,27)^ was done through online meetings with parents, where the researchers could see them applying the program goals and offer simultaneous feedback.

In studies with asynchronous programs^(23,24,28)^, constant feedback was provided through an interactive tutorial^(23)^, while in the other two studies^(24,28)^, parents recorded videos and received feedback on the material.

Therefore, it becomes clearer that combining both methodologies offers more possibilities for the parents' learning process. A study on online parent guidance^(31)^ provided evidence that the combination of these settings is an indicator of satisfaction and learning and a factor impacting family involvement.

Another study^(26)^ on telehealth parental programs found that online therapist assistance is a significant predictor of parents' adherence gains in the intervention. A point found in this review ^(32)^ was the relationship between parents' depressive symptoms and program completion.

Among the experimental studies^(23-25,28)^ found in this review, only one family did not complete the program. This family was in a mixed modality program and did not complete it due to technology issues. However, most studies in this review mentions as a limitation the lack of prior information about the families and the difficulty in measuring the participation time of families. This highlights the importance of analyzing family's profiles to understand the best intervention approach.

Another point that needs to be analyzed is the sociodemographic situation of the families. Given the high cost of in-person interventions, access to the internet and handling technological equipment can be a challenge for family participation in distance interventions. Several studies^(33-35)^ discuss the association between sociodemographic factors and the use of health information technology; however, there was no clear relationship between socioeconomic profile and program retention.

A factor that may impact implementation from the service provider's perspective is the costs involved with telecommunications equipment. However, in the long term, telemedicine could dramatically reduce overall healthcare costs due to its potential to enable a fundamental restructuring of how healthcare is delivered. In countries like Brazil, with vast geographical areas, access to interventions in peripheral, inland, and rural regions is challenging. Therefore, it is necessary to create intervention alternatives that reduce costs, reach more people, and are effective in children's development. The potential of these tools to increase care resolution, shorten distances and isolation between care levels, and reducing referrals and health inequities is essential for regions of the country further from major centers ^(36)^.

Another factor in family engagement and adherence are programs that address skills that directly impact family dynamics. Some research on parental training shows that parents need guidance to understand and stimulate aspects of communication, difficult behaviors, and daily living activities^(37)^. The studies found in this review focused on these themes, with three of them targeting children's communication skills, one on daily living activities, and another on inflexibility behaviors. Addressing topics that impact the family's daily life is crucial for adherence and retention in the intervention program.

Regarding parental engagement, the acquisition of communication and language skills by children with ASD were valid predictors of parents' emotions or attitudes, demonstrating that work focused on these skills can improve parent-child relationship. The studies that targeted communication showed gains in children's skills, such as increased social response, increased requests, improved phrase length, increased communicative intention, increased communicative exchanges with parents, and expanded expressive vocabulary. In the study^(23)^ that addressed daily living activities, there was a decrease in problem behaviors during these tasks and an increase in communication. In the study^(25)^ focusing on inflexibility, there was a decrease in these behaviors.

Indirect intervention, applied by families, gives parents a sense of empowerment, greater involvement in the therapeutic process, and helps reduce stress levels^(38-40)^.

## CONCLUSION

This integrative review on the impacts of distance educational programs for caregivers of children with Autism Spectrum Disorder (ASD) demonstrated that remote parent training can be an effective means of improving parents' knowledge about ASD and their intervention skills. The reviewed studies showed that these programs can enhance the social behaviors and communication skills of children with ASD.

Additionally, this review contributes to the existing literature by consolidating the importance and efficacy of distance parental guidance for early intervention in children with ASD. Despite the diversity of approaches and methodologies, distance educational programs for parents/caregivers have proven to be a valuable tool for improving parental adherence to intervention and the development of children. Future research should continue to explore and refine these interventions, taking into account the individual characteristics and specific needs of the families involved.
