# Supplementary material for: Effectiveness of Distance Educational Programs for parents of children diagnosed with Autism Spectrum Disorder: an integrative review
Source: Codas. 2024 Sep 2;36(5):e20230291. doi: 10.1590/2317-1782/20242023291en (PMC11404835; doi:10.1590/2317-1782/20242023291en)
Supplement: Translation

# Translation: Eficácia de Programas Educativos à Distância para pais de crianças com diagnóstico de Transtorno do Espectro Autista: uma revisão integrativa

**DOI:** 10.1590/2317-1782/20242023291pt

**Authors:** Jullie Gottschall Lima Andrade, Andreia Cristina Feitosa do Carmo, Ana Carina Tamanaha, Jacy Perissinoto

**Affiliations:** 1 Departamento de Fonoaudiologia, Escola Paulista de Medicina, Universidade de São Paulo – UNIFESP - São Paulo (SP), Brasil.; 2 Programa de Pós-graduação Distúrbios da Comunicação Humana, Universidade de São Paulo – UNIFESP - São Paulo (SP), Brasil.; 3 Núcleo de Investigação Fonoaudiológica da Linguagem da Criança e Adolescente no TEA, Escola Paulista de Medicina, Universidade de São Paulo – UNIFESP - São Paulo (SP) , Brasil.

**Keywords:** Autismo, Telemedicina, Desenvolvimento Infantil, Pais, Transtorno do Espectro Autista

## Abstract

**Objetivo:**

analisar a produção científica sobre programas educativos à distância para pais/responsáveis no desenvolvimento de crianças com Transtorno do Espectro Autista (TEA).

**Estratégia de pesquisa:**

utilizou-se a estratégia PICO para identificar o problema de pesquisa. Foram consultadas as bases de dados Medline, ERIC, LILACs, EMBASE, CINAHL, Web of Science e Scopus utilizando descritores e termos livres específicos. não houve restrição de tempo ou idioma. Foram selecionados artigos sobre programas educativos online destinados a pais de crianças com TEA, com foco no impacto desses programas no desenvolvimento de crianças de até seis anos.

**Critérios de seleção:**

os estudos foram selecionados com base em critérios de elegibilidade padrões, incluindo a leitura completa dos artigos após a triagem inicial realizada com o software RAYYAN. Foram incluídos estudos primários, como ensaios clínicos e revisões sistemáticas que avaliaram programas educativos à distância para pais de crianças com TEA.

**Análise dos dados:**

utilizou-se o software RAYYAN para a seleção inicial dos estudos. Os artigos foram organizados hierarquicamente com base no título e resumo, seguido pela leitura integral para aplicação dos critérios de elegibilidade.

**Resultados:**

a busca inicial resultou em 1019 artigos, dos quais 192 foram identificados como duplicados. Após a seleção inicial e a leitura completa, 37 artigos foram analisados, dos quais seis foram considerados elegíveis para responder à pergunta de pesquisa. Entre os estudos elegíveis, um era uma revisão sistemática e cinco eram estudos experimentais. Os estudos experimentais destacaram impactos positivos em áreas como rotinas diárias, flexibilidade comportamental e comunicação. A revisão sistemática indicou evidências preliminares de que os programas educativos à distância para pais podem melhorar o conhecimento sobre TEA, aumentar a adesão às intervenções e promover o desenvolvimento de habilidades sociais e de comunicação nas crianças.

**Conclusão:**

os resultados sugerem que programas remotos de orientação para pais podem ser eficazes para melhorar o conhecimento sobre TEA, aumentar a adesão dos pais às intervenções e promover o desenvolvimento de habilidades sociais e de comunicação em crianças com TEA.

## INTRODUÇÃO

O Transtorno do Espectro Autista (TEA) é uma condição neurológica que se manifesta na infância e perdura ao longo da vida. Caracterizado por déficits persistentes na interação e na comunicação social, comprometimento nas áreas de reciprocidade socioemocional e padrões restritos e repetitivos de comportamento, interesse ou atividades^(1)^. É um transtorno complexo, pervasivo e heterogêneo^(2)^, ou seja, atinge várias áreas do desenvolvimento e apresenta características extremamente variáveis entre os indivíduos acometidos. Esta variabilidade é devida, em parte, à ampla gama de genes e fatores ambientais^(3)^ que podem contribuir para o desenvolvimento do TEA**.** A complexidade desse transtorno representa um desafio significativo para indivíduos, famílias e sistemas de saúde.

Devido ao caráter complexo do TEA é vital a intervenção precoce^(4)^, multidisciplinar e duradoura. A intervenção precoce envolve uma combinação de terapias especializadas, incluindo terapia comportamental, terapia fonoaudiológica, terapia ocupacional e, em alguns casos, medicação^(5)^.

Devido à grande heterogeneidade das crianças com TEA, é necessário optar por intervenções que façam sentido para cada indivíduo e adequar as expectativas de tratamento, pois as respostas ao tratamento podem variar consideravelmente. Segundo o CDC^(6)^, uma em cada 36 crianças de 8 anos nos Estados Unidos é diagnosticada com autismo, o que representa 2,8% da população infantil. Esta alta prevalência apresenta desafios significativos para a rede de saúde e para as famílias, dada a falta de profissionais capacitados e a concentração dos centros de referência em regiões centrais. Em países de grande extensão geográfica, como o Brasil, o acesso a tratamentos e intervenções pode ser particularmente desafiador em regiões periféricas, interiores e zonas rurais^(7)^. Isso destaca a necessidade de criar alternativas de intervenções que sejam não apenas eficazes, mas também acessíveis e escaláveis.

No campo da Fonoaudiologia, a prática da Telefonoaudiologia foi regulamentada com a RESOLUÇÃO CFFa nº 580, de 20 de agosto de 2020. Incluindo prática síncronas e assíncronas, como também teleconsultas, telemonitoramento e oferecendo base para as práticas de orientação parental a distância com uso de tecnologias.

A intervenção precoce, embora benéfica, pode ser onerosa para as famílias e para o Estado, tornando-se necessário criar diferentes formas de intervenção. Estudos recentes têm mostrado a eficácia do uso da tecnologia para a realização de intervenções à distância^(8)^, o que pode ajudar a preencher essa lacuna. No entanto, é importante notar que as modalidades de intervenção à distância podem beneficiar desproporcionalmente aqueles com maior nível socioeconômico, destacando a necessidade de garantir a equidade no acesso a esses recursos^(9)^.

Entre as modalidades de intervenção à distância, a educação e o treinamento dos pais têm demonstrado ganhos importantes no desenvolvimento da linguagem das crianças com TEA. A literatura traz evidências^(10-12)^ que dificuldade ou atrasos na comunicação das crianças estão associados a mudanças de comportamento dos pais.

Com a constante evolução da tecnologia, programas educativos online para pais e responsáveis podem oferecer módulos de aprendizagem interativos, recursos de suporte e orientações de especialistas para abordar questões específicas relacionadas ao TEA^(13)^. Esses programas podem variar desde treinamentos sobre técnicas comportamentais específicas até orientações sobre como navegar nos sistemas de educação e saúde para garantir os direitos e serviços adequados para a criança. No entanto, os benefícios potenciais desses programas devem ser pesados contra os desafios associados. Por exemplo, algumas famílias podem ter dificuldade de acesso à internet confiável ou não estar familiarizadas com a tecnologia necessária para participar desses programas. Além disso, embora muitos programas online sejam projetados para serem autoguiados, alguns pais podem se sentir sobrecarregados sem o suporte direto de um profissional^(14)^.

Dada a diversidade de experiências e contextos dos indivíduos com TEA e suas famílias, é fundamental que os programas educativos à distância sejam flexíveis e personalizáveis para atender às necessidades específicas de cada família. Além disso, as pesquisas devem continuar avaliando a eficácia desses programas em diferentes populações e contextos para garantir que eles sejam uma ferramenta útil e acessível para apoiar o desenvolvimento de crianças com TEA.

Diante disto, se faz necessário compreender e sintetizar o estado atual do conhecimento científico sobre o impacto e compreensão da eficácia e aplicabilidade de programas educativos à distância para pais e responsáveis de crianças com diagnósticos de TEA. Com base nos resultados, recomendaremos estratégias para aprimorar a implementação desses programas, garantindo que beneficiem de maneira mais ampla e equitativa todas as famílias que enfrentam os desafios associados ao TEA.

## MÉTODO

Este trabalho consiste em uma revisão integrativa da literatura^(15)^, um método que permite a combinação de estudos empíricos e teóricos na busca por evidências mais amplas sobre um tópico específico. Devido à natureza inclusiva deste tipo de revisão, é possível abordar uma gama mais ampla de propósitos, como definição de conceitos, revisão de teorias, revisão de evidências e análise de práticas metodológicas^(16)^.

Nossa revisão integrativa seguiu um procedimento estruturado em seis etapas^(17)^: (1) preparação da pergunta de investigação; (2) definição dos descritores e palavras-chave; (3) seleção dos artigos de acordo com os critérios de elegibilidade; (4) coleta, extração de dados, leitura e análise crítica dos artigos; (5) interpretação e discussão dos resultados; (6) síntese do conhecimento e apresentação da revisão^(18)^.

Para formular a pergunta orientadora, recorremos à estratégia PICO^(17,19,20)^ (Paciente, Intervenção, Comparação, Resultados), uma ferramenta útil para estruturar perguntas de pesquisa em saúde. Neste caso, o primeiro elemento (P) referiu-se aos responsáveis ou familiares de crianças autistas; o segundo (I), aos programas educativos à distância para esses responsáveis; o terceiro elemento (C) não foi utilizado nesta revisão; e o quarto elemento (O) diz respeito às habilidades, desenvolvimento da linguagem e cognitivo das crianças.

Assim, a pergunta orientadora desta revisão foi: "Quais os impactos dos programas educativos à distância para responsáveis ou familiares de crianças diagnosticadas com Transtornos do Espectro Autista?"

A busca por estudos relevantes foi realizada em dezembro de 2021 nas bases de dados Medline, ERIC, LILACs, EMBASE, CINAHL, Web of Science e Scopus. Utilizamos uma combinação de palavras-chave e descritores selecionados nas Medical Subject Headings (MeSH), Emtree Terms e nos Descritores em Ciência da Saúde (DeCS) da Biblioteca Virtual em Saúde (BVS) (Tabela 1).

**Tabela 1:** Estratégia de busca para as bases de dados

BASE	ESTRATÉGIA	Qt.
PUBMED	("parents"[MeSH Terms] OR ("caregiver s"[All Fields] OR "caregivers"[MeSH Terms] OR "caregivers"[All Fields] OR "caregiver"[All Fields] OR "caregiving"[All Fields]) OR "parents"[Text Word] OR "family relations"[MeSH Terms] OR "single mother"[Text Word]) AND ("autism spectrum disorder"[MeSH Terms] OR "autistic disorder"[MeSH Terms]) AND ("education, distance"[MeSH Terms] OR ("education"[All Fields] AND "distance"[All Fields]) OR "distance education"[All Fields] OR ("distance"[All Fields] AND "education"[All Fields]) OR "e-learning"[All Fields] OR ("internet"[MeSH Terms] OR "internet"[All Fields] OR "internet s"[All Fields] OR "internets"[All Fields]) OR ("computability"[All Fields] OR "computable"[All Fields] OR "computating"[All Fields] OR "computation"[All Fields] OR "computational"[All Fields] OR "computations"[All Fields] OR "compute"[All Fields] OR "computed"[All Fields] OR "computer s"[All Fields] OR "computers"[MeSH Terms] OR "computers"[All Fields] OR "computer"[All Fields] OR "computes"[All Fields] OR "computing"[All Fields] OR "computional"[All Fields]) OR "internet based intervention"[MeSH Terms] OR "on line intervention"[Text Word])	221
ERIC	(caregivers OR "family relationship" OR "child parent relation" OR “parenting skills”) AND (autism OR “autistic disorders” OR “autistic children” OR “autism spectrum disorders”) AND (“distance education” OR “distance learning” OR tele-education OR "visual classroom" OR e-learnings OR "internet-based intervention" OR "web-based intervention" OR internet) AND ("parent education") AND adult education	488
EMBASE	('child parent relation'/exp OR 'child parent relation' OR 'child parent relationship' OR 'child parent spatial pattern' OR 'correlation, parent child' OR 'parent child correlation' OR 'parent child relation' OR 'parent child relationship' OR 'parent infant bonding' OR 'parent infant relation' OR 'parent-child relations' OR 'parental role' OR 'parenting' OR 'parent'/exp OR 'biological parent' OR 'parent' OR 'parents' OR 'single parent'/exp OR 'lone parent' OR 'mother, unmarried' OR 'parent, single' OR 'single parent' OR 'unmarried mother' OR 'unwed mother' OR 'family relation'/exp OR 'family relation' OR 'family relations' OR 'caregiver'/exp OR 'care giver' OR 'caregiver' OR 'caregivers' OR 'carer' OR 'carers' OR 'family caregiver' OR 'family caregivers') AND ('autism'/exp OR 'kanner syndrome' OR 'pdd (pervasive developmental disorder)' OR 'autism' OR 'autism spectrum disorder' OR 'autism, early infantile' OR 'autism, infantile' OR 'autistic child' OR 'autistic children' OR 'autistic disorder' OR 'autistic spectrum disorder' OR 'child development disorders, pervasive' OR 'childhood autism' OR 'classical autism' OR 'early infantile autism' OR 'infantile autism' OR 'infantile autism, early' OR 'pervasive child development disorders' OR 'pervasive developmental disorder' OR 'pervasive developmental disorders' OR 'typical autism') AND ('distance learning'/exp OR 'distance education' OR 'distance learning' OR 'education, distance' OR 'tele-education' OR 'teleeducation' OR 'virtual classroom' OR 'virtual education' OR 'e learning'/exp OR 'e-learning' OR 'e-schooling' OR 'electronic education' OR 'electronic educational technology' OR 'electronic learning' OR 'on-line education' OR 'on-line learning' OR 'online education' OR 'online learning' OR 'online schooling' OR 'web-based intervention'/exp OR 'internet-based intervention' OR 'internet-intervention' OR 'online-based intervention' OR 'online-intervention' OR 'web intervention' OR 'web-based intervention' OR 'telehealth'/exp OR 'e-health' OR 'ehealth' OR 'tele-health' OR 'telehealth')	187
LILAC’s	("distance education" OR "educacao a distancia"OR e-leraning OR tele-educac* OR internet OR "web basead intervention" OR "online education" OR "educacao online") AND (autism* OR autisti*) AND (pais OR maes OR parentes OR familia* OR cuidador*)	132
CINAHL	("parent education" OR caregivers OR "family relationship" OR "child parent relation" OR "parenting skills") AND (autism OR "autistic disorders" OR "autistic children" OR "autism spectrum disorders") AND ("distance education" OR "distance learning" OR “tele-education” OR "visual classroom" OR “e-learnings” OR "internet-based intervention" OR "web-based intervention" OR internet)	
Web of Science	("parent education" OR caregivers OR "family relationship" OR "child parent relation" OR "parenting skills") AND (autism OR "autistic disorders" OR "autistic children" OR "autism spectrum disorders") AND ("distance education" OR "distance learning" OR “tele-education” OR "visual classroom" OR “e-learnings” OR "internet-based intervention" OR "web-based intervention" OR internet)	40
SCOPUS	(“parent education" OR caregivers OR "family relationship" OR "child parent relation" OR "parenting skills") AND (autism OR "autistic disorders" OR "autistic children" OR "autism spectrum disorders") AND ("distance education" OR "distance learning" OR “tele-education” OR "visual classroom" OR “e-learnings” OR "internet-based intervention" OR "web-based intervention" OR internet)	123

A elaboração da estratégia de busca contou com a contribuição de uma profissional da informação com vasta experiência na área. Além disso, aderiu-se à recomendação PRESS - Peer Review of Electronic Search Strategies^(21)^ no processo de busca e identificação dos estudos, garantindo assim a qualidade, a sistematização e a transparência do processo de construção da estratégia de busca. Não houve restrição de data, visando a uma compreensão abrangente e histórica do tema.

Como critério de inclusão foram considerados programas educacionais online focados no ensino de pais e/ou responsáveis de crianças com diagnóstico de Transtorno do Espectro Autista TEA com idade inferior a seis anos. Nos critérios de exclusão foram: programas educacionais usados para complementar terapias, aqueles que abordassem intervenção direta para as crianças, e estudos que não discutissem o impacto nas habilidades das crianças após a intervenção. Também foram excluídos programas exclusivos para avaliação de habilidades ou diagnóstico.

Após a busca nas bases de dados, o software *Rayyan* ^(22)^ foi utilizado para gerenciar a seleção dos estudos primários. Para a análise dos dados, as duplicatas foram inicialmente excluídas pelo próprio software. Em seguida, duas avaliadoras independentes conduziram a análise, classificando os artigos como "incluído", "talvez" e "excluído".

Depois da seleção final dos estudos, procedeu-se à extração dos dados. Para garantir a consistência e precisão nesse processo, foi desenvolvido um formulário de extração de dados que incluía informações sobre o autor, ano de publicação, país de origem, objetivo do estudo, desenho do estudo, população-alvo, intervenção (se aplicável), principais resultados e conclusões. Esse processo de extração de dados foi conduzido de forma independente por duas revisoras. Cada uma preencheu o formulário de extração de dados para cada estudo selecionado. Após a extração, ambas as revisoras se reuniram para discutir e resolver quaisquer discrepâncias nas informações extraídas. Em casos de discordância, um terceiro avaliador foi consultado para tomar a decisão final. Os dados extraídos foram então sintetizados e organizados de forma a responder à pergunta de pesquisa. O processo de extração de dados foi essencial para garantir a compreensão completa do conteúdo de cada estudo, para permitir análise comparativa e síntese de todos os estudos incluídos.

## RESULTADOS

A busca inicial identificou 1.192 estudos, dos quais trinta e sete foram selecionados para leitura completa do texto. Após a aplicação dos critérios de elegibilidade, seis estudos foram incluídos para análise, como ilustrado na Figura 1.

**Figura 1:** Fluxograma da seleção dos estudos

Os artigos selecionados foram publicados entre 2017 e 2021, sendo três artigos dos Estados Unidos^(23-25)^, um da Austrália^(26)^, um da Islândia^(27)^ e um do Japão^(28)^. A análise dos resultados foi realizada de forma descritiva, considerando as variáveis de interesse desta revisão. As informações detalhadas de cada estudo estão apresentadas na Tabela 1.

As amostras dos estudos eram heterogêneas, variando de 3 pais (em um estudo de caso único) a 104 pais de crianças com TEA. Em todos os estudos experimentais, foi utilizada uma metodologia online e programas faseados.

Sobre a análise de dados dos estudos experimentais desta pesquisa, brilhante-se que todos estavam divididos em três ou quatro fases, presenciais, online ou mistas. Nas fases presencial e online síncrona a orientação acontecia de forma individualizada com cada família. Além disso, a sequência das fases era muito semelhante, ainda que com terminologias distintas.

Os programas descritos pelos artigos demonstraram impactos positivos no desenvolvimento das crianças. Os artigos elegíveis responderam à pergunta norteadora indicaram que o ensino de pais à distância é uma alternativa viável e efetiva para impactar o desenvolvimento das crianças (Quadro 1).

**Quadro 1:** Dados dos estudos incluídos nessa revisão

**Autor**	**Ano**	**Local**	**Objetivos**	**Participantes**	**Tipo de estudo**	**Método**	Principais desfechos
Guðmundsdóttir et al.^(27)^	2018	Islândia	Desenvolver e avaliar um procedimento de telessaúde eficaz para ganhos de habilidades nas crianças com TEA	3	Experimental de caso único	Misto: presencial e online síncrona	Família 1: treinamento não foi eficaz para aumento da extensão de frases, mas foi eficaz para o aumento da resposta social; Família 2: muito eficaz para resposta social e pedidos; Família 3: eficaz para resposta social e ineficaz para extensão de frases
Ibañez et al.^(23)^	2018	USA	Examinar a eficácia de um tutorial parental interativo baseado na web para melhorar o envolvimento das crianças com TEA nas rotinas diárias	104 = grupo Tutorial (n = 52) ou o grupo Controle (n = 52)	Experimental randomizado controlado	Online - Assíncrona	O Grupo tutorial apresentou ganhos nas rotinas diárias, mudou comportamentos relacionados à rotina e em resultados amplos para filhos e pais, como melhora na comunicação social, melhora nos comportamentos problemas. As crianças e seus pais foram capazes de avançar através dos níveis mais baixos de participação (ou seja, tolerante) e atingiu os níveis mais altos de participação.
Douglas et al.^(24)^	2017	USA	Avaliar o efeito de um treinamento online para pais nas habilidades de comunicação das crianças com TEA	3	Experimental de caso único	Misto: presencial e Online - Assíncrono	Na habilidade de oportunidade de comunicação: efeito médio; Na comunicação: a família 1 e 3 apresentou efeitos médios e a 2 efeitos fortes; Resposta dos pais: família altos efeitos e família 2 e 3 fortes efeitos
Hong et al.^(28)^	2018	Japão	Avaliar a eficácia de um pacote de treinamento online no desenvolvimento de linguagem das crianças com TEA e déficits na comunicação	3	Experimental de caso único	Online - Assíncrona	Uma das famílias desistiu do estudo por dificuldade com a tecnologia; Família 1 - houve um aumento substancial das habilidades de comunicação e Família 2: houve um aumento moderado; nos objetivos específicos de comunicação: Família 1 houve um aumento imediato no número de oportunidades de comunicação oferecidas; Família 2: houve um aumento constante no número de palavras diferentes usadas
Kunze et al.^(25)^	2021	USA	Avaliar a eficácia de um programa de treinamento de pais online para diminuir comportamentos inflexíveis nas crianças com TEA	6	Experimental de caso único	Mista: presencial e online - Síncrona e Assíncrona	A família 1 desistiu por intercorrência devido a pandemia; houve melhora nos comportamentos flexíveis e diminuição dos comportamentos inflexíveis em todas as famílias
Parsons et al.^(26)^	2017	Austrália	Desenvolver e avaliar um procedimento de telessaúde eficaz para ganhos de habilidades nas crianças com TEA	3	Revisão Sistemática	Misto: presencial e online síncrona	Família 1: treinamento não foi eficaz para aumento da extensão de frases, mas foi eficaz para o aumento da resposta social; Família 2: muito eficaz para resposta social e pedidos; Família 3: eficaz para resposta social e ineficaz para extensão de frases

## DISCUSSÃO

O propósito desta revisão integrativa foi identificar e analisar os principais resultados presentes em artigos publicados sobre o impacto de programas educativos à distância para pais/responsáveis no desenvolvimento de crianças diagnosticadas com TEA. Nossa análise demonstrou que a orientação parental é um modelo de intervenção promissor para a intervenção precoce nesse grupo de crianças.

Os seis artigos revisados nesta pesquisa^(23-28)^ corroboram a eficácia da intervenção à distância, observando-se uma adesão significativa dos pais ao programa de intervenção e melhorias no desenvolvimento das crianças.

Dos artigos experimentais, foi possível notar uma diversidade de metodologias, destacando a necessidade de futuras pesquisas para determinar práticas mais sistematizadas visando a facilidade de replicação na prática clínica. Outro ponto que difere entre os programas é a modalidade de entrega do programa de ensino – síncrona ou assíncrona. No entanto, a maioria dos estudos (três dos cinco estudos experimentais) combinaram as duas modalidades.

A revisão sistemática^(26)^, encontrada nesse artigo, traz como resultado que os métodos mais interativos de entrega, com vídeos e contato regular com o terapeuta demonstrammelhoras na adesão, aumentaram as taxas de conclusão e melhoraram a adesão às intervenções mediadas pelos pais. Visto isso, a combinação das modalidades pode ser mais eficaz, pois oferece mais possibilidades para o processo de aprendizagem dos pais.

Os estudos experimentais tiveram como característica comum programas de intervenção faseados, a sequência das fases era muito semelhante, ainda que com terminologias distintas. Dentre as fases dos estudos, a maioria deles contou com uma fase pré-intervenção, a fase do treinamento e a fase pós-intervenção.

A fase de treinamento, caracterizada pelo ensino dos pais para realizar a intervenção com as crianças das habilidades alvos, foi a mais diferente entre os estudos, mas a maioria utilizou o método assíncrono (três dos cinco estudos).

A literatura discute que as vantagens do atendimento assíncrono é a redução do impacto na participação por problemas tecnológicos, como conectividade observados na intervenção síncrona. Esse serviço aumenta o acesso, a qualidade do atendimento e os custos do atendimento. Além disso, a intervenção assíncrona apresenta uma possível solução para a escassez de força de trabalho e uma possível estratégia para melhorar os sistemas de atendimento existentes^(29)^.

No entanto, a vantagem do modelo síncrono é a possibilidade de fornecer o feedback imediato, sugerindo ser mais eficaz na produção de mudanças no comportamento^(30)^. A prática síncrona, nos achados dessa revisão^(25,27)^, foi feita por meio de encontros online com os pais, no qual as pesquisadoras poderiam vê-los aplicando os objetivos dos programas e oferecer feedbacks simultâneos.

E os estudos com programas assíncronos^(23,24,28)^, ofereceriam feedbacks constantes por meio de um tutorial interativo^(23)^ e os outros dois estudos^(24,28)^ os pais gravavam os vídeos e recebiam feedback sobre o material.

Visto isso, fica mais claro que a junção das duas metodologias traz mais possibilidades para o processo de aprendizagem dos pais. Um estudo sobre orientação a pais online^(31)^ ofereceu evidências de uma associação entre essas configurações é um indicador de satisfação e aprendizagem e um fator de impacto para o envolvimento do familiar.

Outro estudo^(26)^ sobre programas parentais por telessaúde teve como achado que a assistência online dos terapeutas é um preditor significativo de ganhos de adesão dos pais na intervenção. Um ponto encontrado nessa revisão^(32)^ foi a relação de sintomas depressivos dos pais com a conclusão do programa.

Dos estudos experimentais^(23-25,28)^ – achados nessa revisão - apenas uma família não concluiu o programa. Essa família estava inserida em um programa com modalidade mista e não concluiu o programa referindo problemas com a tecnologia. No entanto, a maioria dos estudos dessa revisão referem como limitação a falta de informações prévias sobre as famílias, além da dificuldade de conseguir mensurar o tempo de participação das famílias. Retomando a importância de análise do perfil da família para entender a melhor forma de intervenção.

Outro ponto que precisa ser analisado é a situação sociodemográfica das famílias. Visto que o custo das intervenções presenciais é elevado, mas o acesso a internet e manuseio de equipamentos tecnológicos podem ser um desafio para a inserção e permanência da família na intervenção a distância. Diversos estudos^(33-35)^ discutem a associação entre fatores sociodemográficos e o uso de tecnologia de informação em saúde, no entanto não houve uma relação clara entre o perfil socioeconômico e a permanência nos programas.

Um fator que pode impactar a implementação, do ponto de vista do prestador de serviço, são os custos envolvidos com equipamentos de telecomunicações.

Mas, a longo prazo, a telemedicina poderia dramaticamente reduzir os custos gerais dos serviços de saúde devido a seu potencial para permitir uma reestruturação fundamental da forma como os cuidados de saúde são prestados. Em países como o Brasil, de grande extensão geográfica, há uma dificuldade de acesso às intervenções em regiões periféricas, interiores e zonas rurais, por isso se faz necessário criar alternativas de intervenções que diminuam os custos, alcancem mais pessoas e sejam eficazes no desenvolvimento das crianças. O potencial dessas ferramentas em aumentar a resolubilidade da atenção, encurtar distâncias e isolamentos entre níveis de atenção, e reduzir encaminhamentos e iniquidades em saúde é essencial para regiões do país mais afastadas dos grandes centros^(36)^.

Outro fator de engajamento e adesão das famílias são programas que abordam habilidades que impactam diretamente na dinâmica familiar, algumas pesquisas feitas sobre treino parental mostram que os pais gostariam de receber orientação para entender e estimular os aspectos da comunicação, comportamentos difíceis e atividades de vida diária^(37)^. E os estudos encontrados nessa revisão focaram nesses temas, sendo que três deles tinham como alvo habilidades de comunicação das crianças, um na rotina de atividade de vida diária e um outro os comportamentos de inflexibilidade. Abordar temas que impactam o cotidiano da família é de extrema importância para adesão e permanência no programa de intervenção.

Dos pontos de engajamento parental, a aquisição das habilidades de comunicação e de linguagem pelas crianças com TEA foram preditores válidos das emoções ou atitudes dos pais, demonstrando que o trabalho direcionado para essas habilidades pode melhorar a relação pai e filho. Os estudos que tiveram como meta a comunicação mostraram ganhos de habilidades nas crianças, como: aumento da resposta social, aumento dos pedidos, melhora na extensão de frases, aumento da intenção comunicativa, aumento da troca comunicativa com os pais e ampliação do vocabulário expressivo. No estudo^(23)^ que abordou atividade de vida diária, houve diminuição dos comportamentos problemas durante essas tarefas e aumento da comunicação. No estudo^(25)^ que focalizou a inflexibilidade, houve diminuição desses comportamentos.

A intervenção indireta, aplicada pelas famílias, dá aos pais a sensação de empoderamento, maior pertencimento ao processo terapêutico e auxilia na diminuição dos níveis de estresse^(38-40)^.

## CONCLUSÃO

Esta revisão integrativa sobre os impactos dos programas educativos à distância para responsáveis de crianças com Transtornos do Espectro Autista demonstrou que o treinamento de pais entregue remotamente pode ser um meio eficaz de melhorar o conhecimento dos pais sobre TEA e suas habilidades de intervenção. Os estudos revisados mostraram que esses programas podem ampliar os comportamentos sociais e as habilidades de comunicação das crianças com TEA.

Além disso, essa revisão contribui para a literatura existente ao consolidar a importância e a eficácia da orientação parental à distância para a intervenção precoce em crianças com TEA. Apesar da diversidade de abordagens e metodologias, os programas educativos à distância para pais/responsáveis demonstraram ser uma ferramenta valiosa para aprimorar a adesão dos pais na intervenção e o desenvolvimento das crianças. Futuras pesquisas devem continuar a explorar e aprimorar essas intervenções, levando em consideração as características individuais e as necessidades específicas das famílias envolvidas.
